# Hepatic perivascular epithelioid cell tumor: a retrospective analysis of 36 cases

**DOI:** 10.3389/fonc.2024.1416254

**Published:** 2024-08-16

**Authors:** Min Ji, Yuchen Zhang, Shuaibing Liu, Menghui Zhang, Bingbing Qiao

**Affiliations:** Department of Hepatobiliary and Pancreatic Surgery, The First Affiliated Hospital of Zhengzhou University, Zhengzhou, China

**Keywords:** abdominal tumor, liver, PEComa, diagnosis, treatment

## Abstract

**Background and aims:**

Hepatic perivascular epithelioid cell tumor (PEComa) is a rare type of mesenchymal neoplasm and lacks systematic reports. The aim was to analyze the features of hepatic PEComa in order to provide our own experience for diagnosis and management from a single center.

**Methods:**

We retrospectively analyzed clinical data, imaging findings, pathology, treatments and prognosis of 36 patients with hepatic PEComa in the First Affiliated Hospital of Zhengzhou University from January 2016 to September 2023.

**Results:**

29 females and 7 males (median age, 47.8 years) were included in this study. The majority (26/36, 72.2%) of patients were diagnosed incidentally with non-specific symptoms. Abnormal enhancement of enlarged blood vessels (27/36,75%) can be observed on CT/MRI and only 7 patients (19.4%) were correctly diagnosed by imaging examinations. The positive immunohistochemical expressions were HMB-45(35/36,97.2%), Melan-A (34/35,97.1%), SMA (23/26,88.5%) and CD34(86.7%,26/30). Treatments include resection (24/36,67.7%), radiofrequency ablation (6/36,16.7%), transcatheter arterial chemoembolization(1/36,2.7%), conservative clinical follow-up(2/36,5.6%), and sirolimus-chemotherapy (3/36,8.3%). During the follow-up period (range, 2–81 months), except for one patient with a single intrahepatic recurrence and 3 malignant patients died in 6 months, the remaining patients had no signs of recurrence and metastasis.

**Conclusions:**

Hepatic PEComa has no specific clinical features and mainly depends on clinicopathological characteristics for accurate diagnosis. Resection is the best treatment for benign PEComa, but TACE and radiofrequency ablation can also be considered in case of contraindications for surgery.

## 1 Introduction

Perivascular epithelioid cell tumors (PEComas) are a rare type of neoplasms originating from mesenchymal tissues and initially proposed by Bonetti et al (1) in 1992. In 2002, the World Health Organization (WHO) introduced a new classification category for PEComas, defining them as abnormal mesenchymal neoplasia characterized by perivascular epithelioid cells exhibiting distinctive histological and immunohistochemical features (2). This family of tumors includes angiomyolipoma (AML), lymphangioleiomyomatosis (LAM), pulmonary clear cell “sugar” tumors and PEComa-not otherwise specified (PEComa-NOS) which can occur in gastrointestinal, gynecologic, genitourinary and other organs with low incidence (3–6). Hepatic PEComa is extremely rare with no specific symptoms and mostly reported as individual cases (7). The limited understanding of this hepatic lesion poses challenges in distinguishing it from other liver tumors, such as hepatocellular carcinoma (HCC), focal nodular hyperplasia (FNH), hepatic hemangioma and hepatocellular adenoma (8–11). In this study, we retrospectively analyzed the clinical data, imaging findings, pathological features, immunohistochemical phenotypes, and treatment modalities of 36 cases with hepatic PEComa in our center. And the effects of different methods on diagnosis and prognosis were evaluated to provide information for the guidance of clinical treatment. This report is the largest single-center study of hepatic PEComa covering different treatments to date.

## 2 Materials and methods

### 2.1 Patients selection

This was a retrospective observational study in the 1st Affiliated Hospital of Zhengzhou University. We collected all eligible case data of 42 patients with hepatic PEComa from January 2016 to September 2023. Patients meeting the following criteria were included (1): adult (age≥18 years old) (2); pathologically diagnosed with hepatic PEComa (3); patients received no related treatment for the disease prior to hospitalization and no additional treatment for other malignancies during the same period of hospitalization (4); regularly followed up and with complete follow-up data. This study received approval from the institutional ethical review board, and informed consent was obtained from all patients or their guardians.

### 2.2 Clinical data and imaging representation

Clinical data for all patients were adequately recorded including age, sex, size and location of tumor, clinical presentation and history, routine blood test, liver function tests, hepatitis virus antigen and tumor-specific markers. Each patient received at least one or multiple imaging examinations, such as abdominal ultrasound, contrast-enhanced ultrasound (CEUS), computer tomography (CT)/magnetic resonance imaging (MRI) or 18F-fluorodeoxyglucose positron emission tomography/CT (18-FDG PET/CT).

### 2.3 Pathology and immunohistochemistry

The tumor specimens were routinely fixed with 4% formaldehyde, embedded in paraffin and sectioned. Immunohistochemical stains including human melanoma black 45 (HMB-45), Melan-A, smooth muscle actin (SMA), S-100, CD34, Desmin, TEF-3, Ki-67, alpha-fetoprotein (AFP), Pancytokeratin 8(CK8), Panytokeratin 18(CK18) and hepatocyte paraffin 1 (Hep-Par1), were applied for differential diagnosis. Two pathologists reviewed the sections, marked the representative regions of tissue blocks, and assessed the histological features.

### 2.4 Follow-up

All patients were followed by telephone or outpatient review, and the follow-up period extended to December 2023. During the first year of follow-up, patients were scheduled for at least one follow-up examination within 6 months. The follow-up examinations included ultrasound and liver function tests. If considered necessary, additional CT or MRI were performed. Subsequently patients were required to undergo at least one outpatient review or telephone interview to assess the presence of tumor recurrence and metastasis every year.

### 2.5 Statistical methods

The statistical software SPSS 20.0 (SPSS Inc.) was used for statistical analysis. Continuous values are presented as the mean ± standard deviation (SD) or range, and categorical data are reported as the number of patients (percentage). All statistical p values were two-sided, with p values of <0.05 were considered to indicate statistical significance.

## 3 Results

### 3.1 Clinical characteristics and serum biomarkers

Thirty-six of 42 patients were enrolled in our study; 6 patients were excluded because they were lost to follow-up or had incomplete data. Among them, there were 7 males and 29 females, with an average age of 47.8 ± 12.9 (range 25–78) years. Among all patients, 31 cases (86.1%) had single lesions and 5 cases (13.9%) had multiple lesions. The maximum cross-sectional diameter of the tumor was about 1.7–21.6 cm, with an average of (7.05 ± 5.50) cm, including 22 cases (61.1%) in the right liver, 9 cases (25.0%) in the left liver, 2 cases (5.6%) in the caudate lobe, and 3 cases (8.3%) of extrahepatic metastasis. Of the 36 patients, 8 patients presented with upper abdominal intermittent pain or discomfort, while 26 patients were diagnosed incidentally with non-specific symptoms during the routine physical examination. One patient experienced recurrent low fever due to pulmonary infection and the other one experienced compression pain on the right kidney when the tumor involved into surrounding tissue. 6 patients had a history of other liver disease (cysts or hemangioma). None of the cases was complicated with history of alcohol, smoking, drug or tuberous sclerosis complex (TSC). Regarding the complete blood count (CBC) analysis, 8 patients had anemia of varying degrees. Except for only one patient with positive hepatitis B virus surface antigen, liver functions, a-fetoprotein (AFP), carcinoembryonic antigen (CEA) and carbohydrate antigen 19–9(CA19–9) remained within the normal range. 4 cases had elevated carbohydrate antigen 125(CA125).

### 3.2 Imaging findings

**Figure 1:**
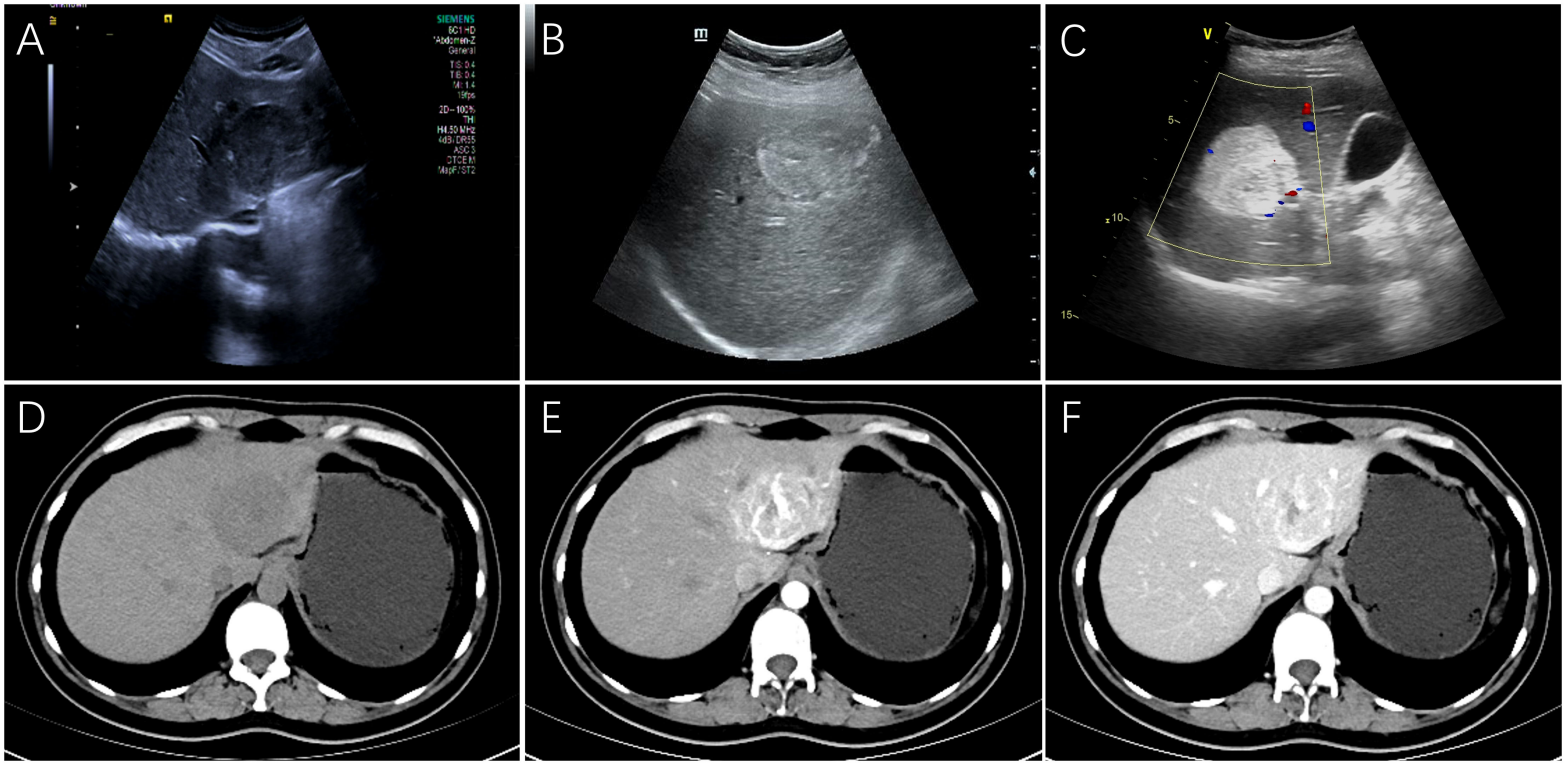
The features on Ultrasonography/dynamic CT of hepatic PEComas. Ultrasonography showed a hypoechoic mass with regular shape **(A)**, a heterogeneous mixed echo mass **(B)** and a hyperechoic mass with clear boundary **(C)** on three different female patients, respectively. On CT scanning of a 40-year-old female patient, the plain scan showed a circular, low-density tumor with well-defined boundaries **(D)**. Markedly inhomogeneous enhancement and abnormally dilated blood vessels can be observed in the arterial phase **(E)**, and the enhancement decreased with washout pattern in the portal vein phase **(F)**. (PEComa, perivascular epithelioid cell tumor; CT, computed tomography).

**Figure 2:**
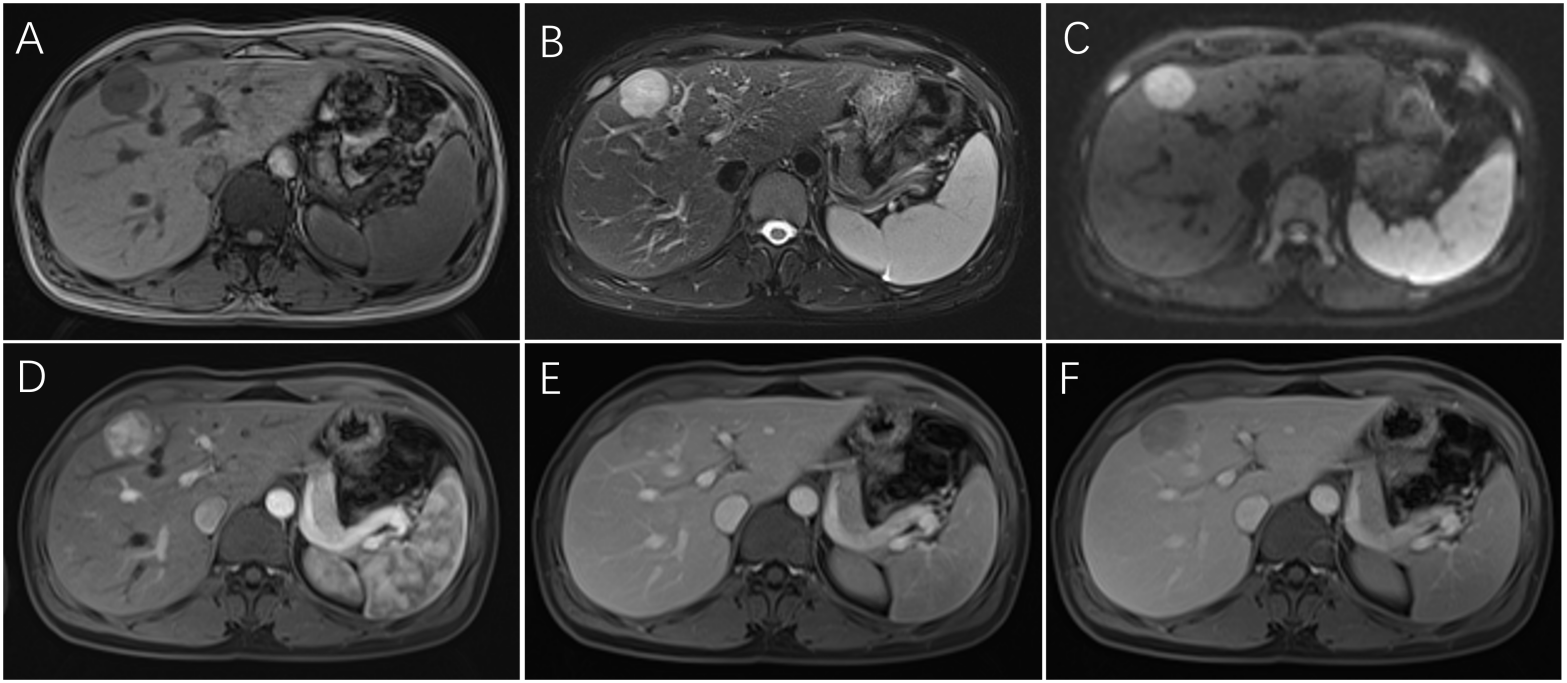
Dynamic MRI of hepatic PEComas. A 33-year-old woman with hepatic PEComa was admitted for the presence of liver mass with non-specific symptoms, who was first misdiagnosed as hepatocellular carcinoma on imagings. MRI showed a regular well-defined mass in segment IV of the liver, with hypointensity on T1-weighted images **(A)**, hyperintensity on fat-suppressed T2-weighted images **(B)**, and hyperintensity on diffusion-weighted images **(C)**. The tumor was significantly heterogeneous enhanced and showed high signal in the arterial phase **(D)**, which decreased rapidly in the portal venous phase with fast-washout pattern **(E)** and had a lower signal in the delayed phase in comparison with the surrounding hepatic parenchyma **(F)**. (PEComa, perivascular epithelioid cell tumor; MRI, magnetic resonance imaging).

Ultrasonography was performed on 32 patients, and the lesions were all solid, 18 cases showed hypoechoic mass, 6 cases showed mixed echo, and 8 cases showed hyperechoic or slightly hyperechoic (**Figures 1A–C**). Among them, 3 patients received additional CEUS of the liver. After the injection of the contrast agent, the tumor in the arterial phase was rapidly enhanced and showed high signal, which was reduced gradually in the portal venous phase and had a weakened enhancement during the delayed phase in comparison with the surrounding hepatic parenchyma. 26 patients received CT scans with low or mixed density during plain scans (**Figure 1D**), and the arterial phase was significantly enhanced. The enhancement was weakened in 23 cases during the portal venous (**Figures 1E, F**), while it still enhanced in the remaining 3 cases. 14 patients received MRI examination, including 3 cases imaged with gadoxetate disodium (Gd-DTPA)-enhanced MRI and 11 cases imaged with gadopentetate dimeglumine (Gd-BOPTA)-enhanced MRI. All of 14 cases showed hyperintensity on diffusion-weighted images (DWI) (**Figure 2C**), and hypointensity on T1-weighted images(T1WI) (**Figure 2A**), while 9 cases were hyperintensity or slightly hyperintensity on fat-suppressed T2-weighted images(T2WI) (**Figure 2B**), and 5 cases were mixed signal. Arterial phase enhancement was obvious in all of 14 cases, the later phase enhancement decreased in 12 cases (**Figures 2D–F**), while it still enhanced in the remaining 2 case. All of 3 patients with Gd-DTPA-enhanced MRI showed low signal on hepatobiliary phase images (HBP). Combining with ultrasonography, CT and/or MRI, the presence of other imaging findings were fat, necrosis, hemorrhage, calcification, cyst and dysmorphic vessels in the tumors. The imaging characteristics on CT/MRI of the 36 patients is summarized in **Table 1**. A 47-year-old female patient with multiple intrahepatic masses underwent 18F-FDG PET/CT examination, which showed a mixed-density mass shadows with active metabolism in the right liver, and SUVmax of about 5.8. Only 7 patients (19.4%) were correctly diagnosed as PEComa by all imaging examinations.

**Table 1:** The imaging characteristics on CT/MRI of the 36 patients.

		Positive cases (n)/total	% Positive
**Number**	1	31/36	86.1
	2	2/36	5.6
	≥3	3/36	8.3
**Size** (cm)	>10	7/36	19.5
	5–10	12/36	33.3
	3–5	10/36	27.7
	≤3	7/36	19.5
**CT**	Low density on plain scans	24/26	92.3
	Mixed density on plain scans	2/26	7.7
	Arterial enhancement with fast washout	15/26	57.7
	Arterial enhancement with slow washout	8/26	30.8
	Arterial enhancement with persistent enhancement in the late phases	3/26	11.5
**MRI**	DWI high SI	14/14	100
	T1WI low SI/Fat-suppressed T2WI high SI	9/14	64.3
	T1WI low SI/Fat-suppressed T2WI mixed SI	5/14	35.7
	Arterial enhancement with fast washout	9/14	64.3
	Arterial enhancement with slow washout	3/14	21.4
	Arterial enhancement with persistent enhancement in the late phases	2/14	14.3
	Hepatobiliary phase images low SI	3/3	100
**Others**	Fat	5/36	13.9
	Necrosis	6/36	16.7
	Hemorrhage	4/36	11.1
	Calcification	1/36	2.8
	Cyst	2/36	5.6
	Dysmorphic vessels	27/36	75.0
**Imaging diagnosis**	HCC	17/36	47.3
	FNH	5/36	13.9
	PEComa	7/36	19.4
	Hepatic hemangioma	4/36	11.1
	Hepatocellular adenoma	3/36	8.3

CT, computed tomography; DWI, diffusion-weighted imaging; FNH, focal nodular hyperplasia; HCC, hepatocellular carcinoma; MRI, magnetic resonance imaging; PEComa, perivascular epithelioid cell tumor; SI, signal; T1WI, T1-weighted imaging; T2WI, T2-weighted imaging.

### 3.3 Pathological features and immunohistochemistry

**Figure 3:**
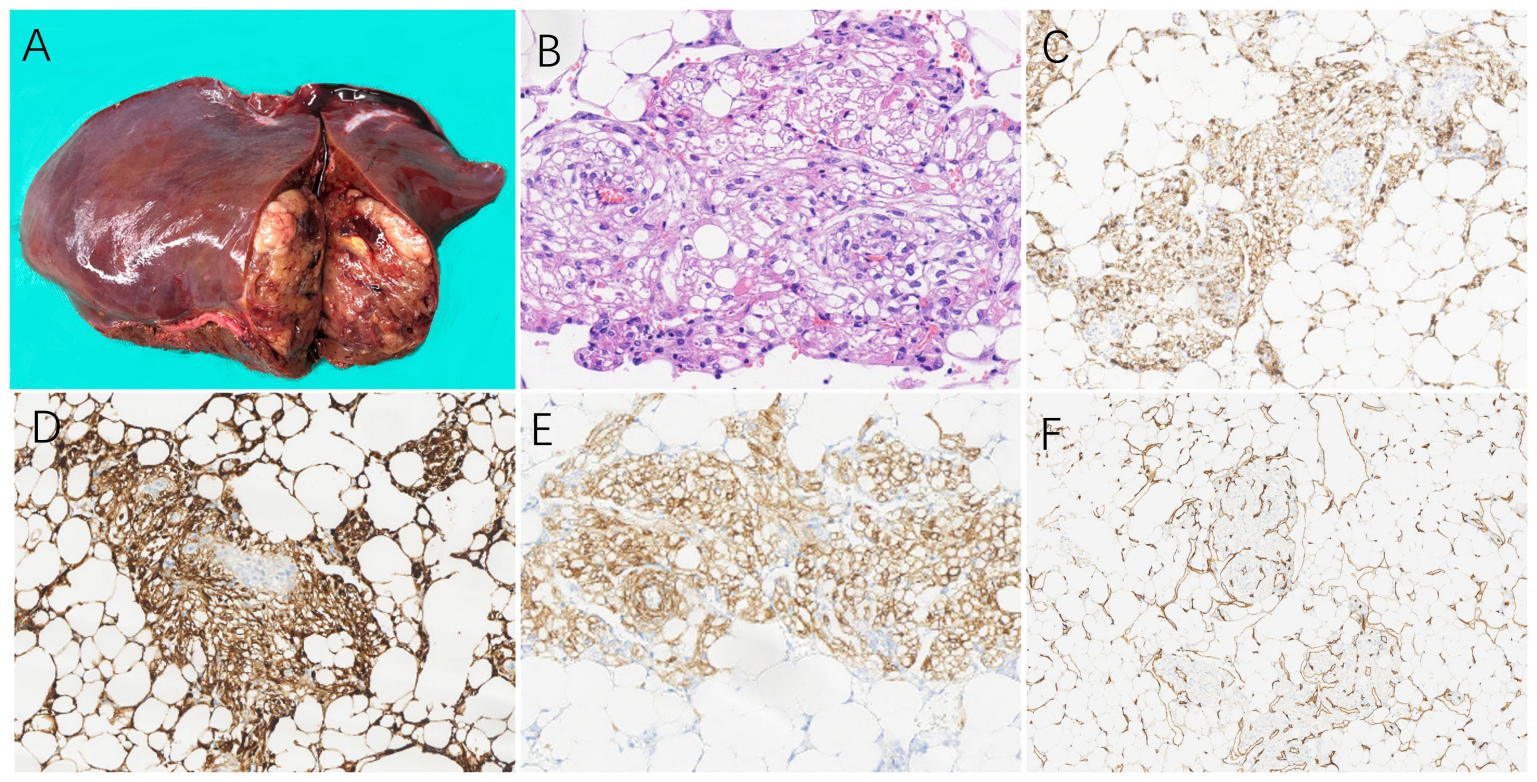
The pathological and immunohistochemical characteristics on hepatic PEComas. **(A)** On a 49-year-old female patient, the specimen section after resection on the left liver showed grayish yellow on the cut surface with soft texture and clear boundary. Microscopically, **(B)** epithelioid cells were arranged in in wheel pattern with cytoplasm ranging from eosinophilic granular to transparent, and the mature lipocytes scattered in distribution. (H&E staining, 200×). Immunohistochemistry showed the positive expressions of the tumor cells were **(C)** HMB-45 (magnification × 100), **(D)** Melan A (magnification × 100), **(E)** SMA (magnification × 100), and **(F)** CD34 (magnification × 200). (PEComa, perivascular epithelioid cell tumor; H&E, haematoxylin and eosin; HMB-45,human melanoma black 45;SMA, smooth muscle actin).

Prior to initiating treatment, liver biopsy was performed on 13 patients to establish a definitive diagnosis for hepatic PEComa. Among them, 24 underwent surgical resection and obtained gross specimens. The tumors were solid with a slightly soft or moderate texture and the cut surface was grayish yellow or dark red without obvious capsule (**Figure 3A**). Areas of hemorrhage were present in 3 cases. 21 cases were clearly demarcated from the adjacent hepatic parenchyma while the other 3 cases were ill-defined. Histologically, the tumor cells of PEComas were mainly composed of epithelioid cells, spindle cells and eosinophilic cells. The majority of patients were comprised of perivascular cells with a small account of adipocyte less than 5% or none. And the tumor cells were polygonal or spherical in shape with distinct cell borders, characterized by abundant cytoplasm ranging from eosinophilic granular to transparent (**Figure 3B**). 3 cases were diagnosed as classical epithelioid angiomyolipoma (CAML), which showed smooth muscle cells were arranged in whorled around by thick-walled blood vessels with the mature adipose tissue. In addition, extramedullary hematopoiesis can be observed in 8 cases. The mainly positive rates of immunohistochemical staining for various tumor markers were 97.2% (35/36) for HMB-45, 97.1% (34/35) for Melan-A, 88.5% (23/26) for SMA, and 86.7%(26/30) for CD34 (**Figures 3C–F**). Additionally, the neoplastic cells were all negative for AFP, CK8, CK18 and Hep-Par1. Except for 4 tumors whose Ki-67 staining reached more than 20%, the Ki-67 staining was lower than 10% in other tumors. The general information about pathological features and immunohistochemical staining is summarized in **Table 2**.

**Table 2:** The pathological features and immunohistochemical staining in the 36 patients.

			Positive cases (n)/total	% Positive
Pathological features		Benign	33/36	91.7
		Malignant	3/36	8.3
Texture		Soft	17/24	70.8
		Moderate	7/24	29.2
Boundary		Clear	21/24	87.5
		Unclear	3/24	12.5
Color of cut surface		Grayish yellow	14/24	58.3
		Dark red	10/24	41.7
Immunohistochemistry				
HMB-45			35/36	97.2
Melan-A			34/35	97.1
SMA			23/26	88.5
CD34			26/30	86.7
S-100			7/33	21.2
Desmin			5/16	31.3
TFE-3			5/15	33.3
Ki-67	≤5		29/36	80.6
	5–10		3/36	8.3
	>10		4/36	11.1

HMB-45, human melanoma black 45; SMA, smooth muscle actin;

### 3.4 Therapeutic methods and follow-up

**Figure 4:**
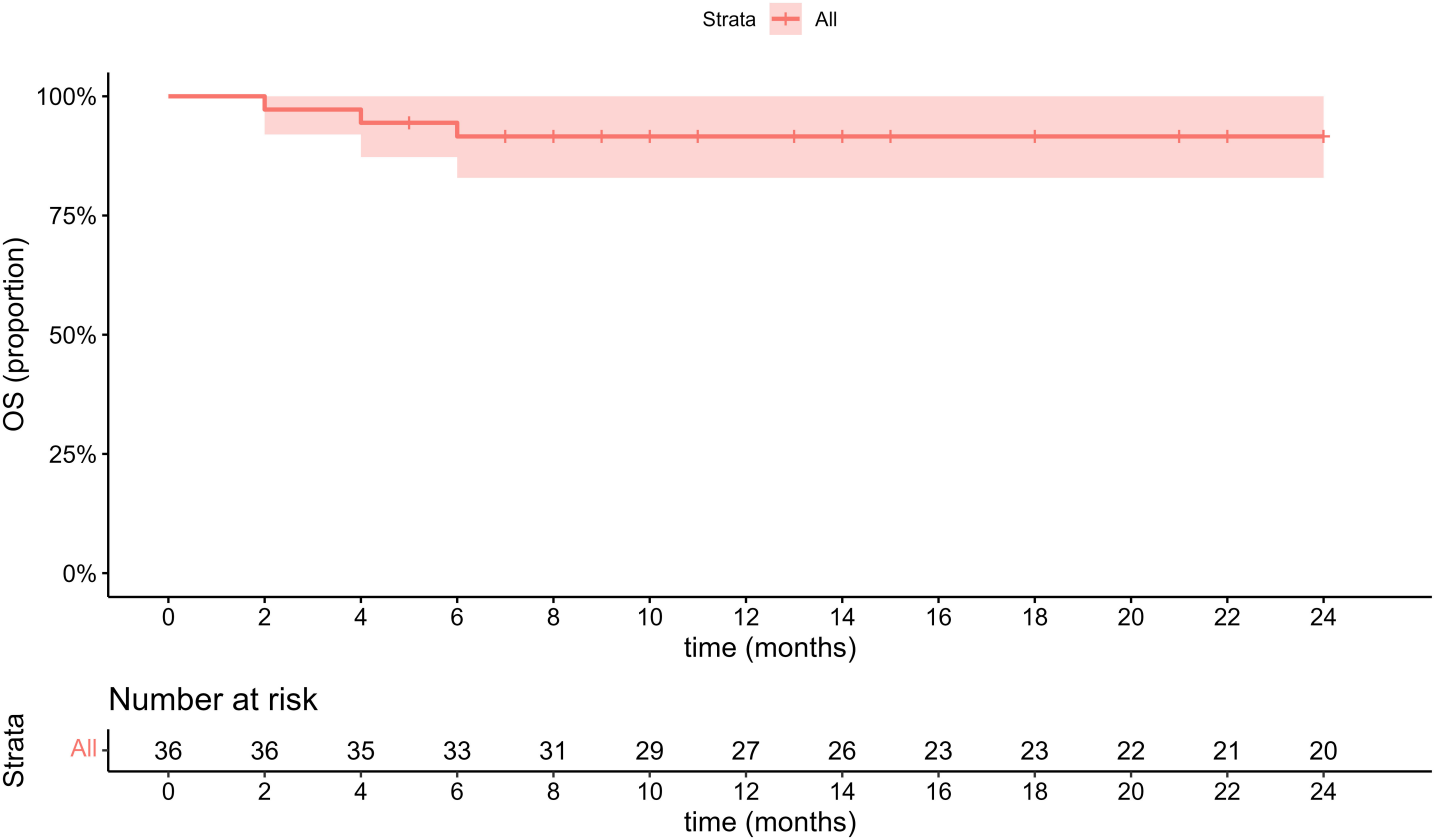
2-year overall survival in the entire population.

The treatment plan consisted of various approaches: 13 cases underwent open surgical resection and 11 cases underwent laparoscopic resection (complete resection of tumors was guaranteed in all operations), 2 cases were placed under conservative clinical follow-up, 6 case underwent radiofrequency ablation, 1 case with a size of about 14.6 cm involving cavernous transformation of the portal vein (CTPV) underwent transhepatic arterial chemoembolization (TACE) twice within 3 months due to inoperability, and 3 cases with malignant PEComas received sirolimus combining with chemotherapy. The median follow-up time was 35.5 ± 26.4 (range 2–81) months. 3 patients with malignant PEComas died in the 2nd, 4th and 6th month of follow-up respectively while 1 case died of massive hemorrhage due to tumor rupture, and the other 2 cases died of multiple organ failure due to tumor progression (**Figure 4**). In addition, the only 1 patient treated with TACE was currently alive with tumor in a stable state for 18 months. One case whose tumor size was 12.8 cm with necrosis had a single intrahepatic recurrence 34 months after surgery and underwent a second surgical resection. None of the remaining 31 patients had metastasis or recurrence during the follow-up period. The general information about treatment is summarized in **Table 3**.

**Table 3:** The information about treatment for the 36 patients.

			Cases (n=36)	Gender(M/F)	Age (Mean ± SD, years)	Size (Mean± SD, cm)	P-Value
**Resection**			24 (66.7%)	3/21	47.0 ± 13.2	6.88 ± 4.89	
	Size	<3	3	1/2		2.30 ± 0.50	
		3–5	6	1/5		3.78 ± 0.53	
		5–10	12	0/12		6.74 ± 1.13	
		>10	3	1/2		18.23 ± 3.68	
	Type	Open surgical	13	1/12	47.8 ± 14.0	9.05 ± 5.58	0.012
		laparoscopic	11	2/9	46.2 ± 12.9	4.33 ± 2.11	
**Radiofrequency ablation**			6 (16.7%)	2/4	48.3 ± 13.8	3.33 ± 0.60	
**Conservative**			2 (5.6%)	1/1	52.5 ± 14.8	2.05 ± 0.50	
**TACE**			1 (2.7%)	0/1	51	14.6	
**Sirolimus/chemotherapy**			3 (8.3%)	1/2	48.0 + 16.5	14.8 ± 3.40	

TACE, transhepatic arterial chemoembolization.

## 4 Discussion

Hepatic PEComa is a rare mesenchymal tumor while PEComa most commonly occurs in the kidney and uterus (3, 12, 13). Krawczyk Met al (7)reported hepatic PEComa tend to occur in females, who accounted for 80.6% of patients in our study. Of 36 patients, we found no association with alcohol, smoking, drugs, hepatitis, or other liver disease. It was reported that hepatic PEComa is partially associated with a genetic disease called TSC in a small percentage of cases, because the pathogenesis of PEComa may be related to the deletion of TSC1 or TSC2 (6, 14). However, none of the patients reported in this study had a history of similar hereditary diseases in their family, which may be limited by the small number of cases. Further studies are warranted to investigate the pathogenesis of hepatic PEComa. Patients with PEComas usually have non-specific symptoms (11, 13). In our study, the majority(72.2%) of patients detected space-occupying lesions in the liver by accidental physical examination without obvious clinical symptoms. When the tumor grew to compress the surrounding tissue, the patients partly (22.2%) had experienced abdominal pain or discomfort. In addition, compared with previous literature reports (3, 8, 9, 11, 13), we newly found 8 cases (25%) of the patients had varying degrees of anemia, which may be associated with extramedullary hematopoiesis (7, 15). In terms of laboratory examinations, except for a few patients (4/36) with elevated CA125, other indicators of the patients were normal. Clinically, this type of tumor lacks specific tumor markers and is difficult to diagnose only by laboratory examinations (8).

Imaging examination is one of the most important methods to diagnose for hepatic PEComa. The imaging characteristics are correlated with the variable proportions of the different components such as adipocytes, perivascular cells, and enlarged blood vessels within tumors (8, 16, 17). In our study hepatic PEComas are more likely to occur in the right lobe(61.1%), but Yang et al (18)reported that the number of tumors in the left lobe was approximately equal to the right lobe as the sample size increased. The ultrasound features of tumors are usually circular or quasicircular inhomogeneous solid masses with clear boundaries, showing various echogencities (19, 20). On the CEUS examination, the arterial phase rapidly enhances and shows high signal, which is basically consistent with the results obtained in the patients of our study. And in the late phases, it can fade to a hypoenhancing pattern of slow washout or still show a persistent hyperenhancing pattern, which may be related to the long-term retention of the contrast agent due to the rich capillary network in the tumors (10, 13, 19, 21). On non-enhanced CT and MRI, the tumors show low density on plain CT scan, and low signal intensity on T1WI, high signal intensity on fat suppressed T2WI and DWI (10, 22). However, these features are non-specific in mostly other liver tumors. On contrast-enhanced CT/MRI of hepatic PEComa, the lesions mostly showed enhanced obviously in the arterial phase with washout pattern, who accounted for 86.1% (31/36) of patients in our study and while all of 3 cases with specific hepatic contrast agent showed low signal on MRI hepatobiliary phase images, which were especially easily confused with HCC (10). The specific manifestations can be observed on ultrasound, CT and MRI, which can help to effectively improve the accuracy of diagnosis. First, in our study the abnormal enhancement of enlarged blood vessels can be observed in most (27/36,75%) of tumors, which is the most significant characteristic of hepatic PEComa and different from the vascular shadows of HCC (10, 17, 18). Secondly, although the enhancement pattern of “fast in and fast out” in HCC is similar to hepatic PEComa, the lesions show significant heterogeneous enhancement in the arterial phase due to neovascularization and arteriovenous connections (10, 22). In addition, HCC usually presents aggressive growth with ill-defined borders, cirrhosis, and even bleeding or portal vein emboli. Most benign hepatic PEComas usually have a clear boundary and lack capsule without aggressive behavior, and the presence of adipose tissue in CAML is easy to distinguish particularly on ultrasound and MRI (20, 23). Even combining multiple imaging examinations, the accuracy of diagnosis was only 19.4% in our study, which was close to the rate of 20% reported in other studies (8, 13, 18).

Due to the non-specific clinical presentation and imaging findings, the diagnosis of hepatic PEComa depends more on pathologyl and immunohistochemistry to distinguish the other lesions in the liver (11). By gross observation, the tumors are quasi-circular and moderate in texture, and the cut surface is usually yellow or dark red without obvious capsule. Microscopically, the tumor cells mainly composed with epithelioid cells, spindle cells and eosinophilic cells, which are arranged in sheets or nest radially around dilated blood vessels and often show clear to lightly eosinophilic cytoplasm with central oval nucleus (24, 25). Adipose tissues can also be observed within section of tumors. Immunohistochemically, the positive expression of PEComa include melanocytic markers (HMB 45, Melan-A, S-100), myogenic markers (SMA) and angiogenic markers (CD34), and negative expressions include AFP, Hep-Par1, CD117, CK8/18, and TFE-3 (3, 9, 13, 25). In this study, the positive rates of SMA, HMB45, and Melan-A were all above 85%, but we found that the positive rate of CD34 was significantly higher than that reported in previous articles. The expression of CD34 may be related to the abnormal neovascularization in the tumor. It is recommended to detect the expression of TFE-3 protein, because TFE3-positive PEComas are associated with poor prognosis (3, 26). In our study, we detected positive TFE3 protein in only 5 patients out of 15, and the prognosis of the patients was good except one patient died.

Up to now, the criteria for malignancy and biological behavior of PEComas have not uniformly established. Indeed, Folpe et al (6) have suggested malignant PEComas are characterized by any two of the following criteria: tumor size>5cm, infiltrative growth pattern, high nuclear grade and cellularity, necrosis, vascular invasion, mitotic activity of more than 1/50 high power field(HPF), and aggressive clinical behavior. These criterias were observed in the 3 cases of our study diagnosed malignant PEComas, one of whom was confirmed systemic metastases by 18-FDG PET/CT. Therefore, 18-FDG PET/CT plays an important role in differentiating benign PEComas from malignant tumors and detecting occult metastases (27, 28). In our study, most benign patients (29/33, 87.9%) had tumors <10 cm in size and we should take into account that tumors >10 cm tend to become malignant. Chemotherapy and antiangiogenic agents for malignant PEComa have no remarkable benefits (5, 12, 29, 30). Studies suggested that mTOR inhibitors therapy such as sirolimus may be a viable treatment option with significant clinical responses for patients with malignant perivascular epithelioid cell tumors (27, 31, 32). In a 10-year retrospective study of 15 patients with advanced or metastatic PEComa, 8 of whom underwent surgery for the primary tumor, the benefit of using mTOR inhibitors(sirolimus, 2–6 mg per day) objective response (ORR): 73%(11/15) and progression-free survival(PFS): not-reached (95% CI: 42.0-NA); compared to first-line chemotherapy with ORR: 25%(1/4) and PFS:4.9 months (95% CI: 3.8–NA) (30). It was also recently reported that everolimus has a better therapeutic efficacy than sirolimus, particularly in reducing PEComa subtype, TSC-related AML volume (33). In an open-label, single-arm, phase IIIb trial of TSC-AML, 19 patients were enrolled and started once-daily oral administration of everolimus at a dose of 10 mg for a median of 6.6 (5.3–10.9) months. 11 patients (57.9%) experienced at least a 30% reduction in tumor volume in the first 6 months of treatment with none progressed (34). These studies provided data for the selection of systemic therapy and potential modalities of neoadjuvant and adjuvant therapy in patients with advanced or metastatic PEComa (31–35). During the period of medication, it is necessary to pay attention to whether drug toxicities and adverse reactions, especially dose-dependent non-infectious pneumonitis (36). In our study, all 3 cases of malignant hepatic PEComa with metastases received the above treatments, but died within half a year without significant effect, probably because the 3 patients were already at the end stage of the tumor when they were diagnosed. Due to the rarity of PEComa, the efficacy of other therapies has not been demonstrated by prospective clinical trials or sufficiently large retrospective case series and the vast majority were case reports. A case of unresectable liver PEComa with a 700 cm^3^ lesion in segment IV received stereotactic radiation therapy (SBRT; 8 fractions of 7.5Gy) and remained disease-free for at least 21 months (37). The role of radiotherapy in PEComa is not clear. Therefore, the decision should be individualized for each patient during a multidisciplinary care. At present surgical resection can be the best choice for benign PEComa therapy (13, 25). Except for 1 case of patients with recurrence 34 months after surgery, the other 23 cases who underwent surgical resection had good outcomes. In our study, the size of the tumor is closely related to the type of surgery, whether open or laparoscopic (9.05 ± 5.58 VS. 4.33 ± 2.11cm, P=0.012). We found 2 asymptomatic patients with tumor size of 1.7cm for 15-months followed-up time and 2.4cm for 36-months respectively had no significant progression or metastasis during conservative follow-up period. This indicates that asymptomatic patients with PEComas of <3cm and exclusion of malignancy might be administered conservative treatment. In addition, we performed ultrasound radiofrequency ablation therapy under local anesthesia for 6 patients with tumors less than 5cm in size, who could not tolerate general anesthesia due to cardiopulmonary function or unwilling to surgery.

According to the principle of locoregional therapy in The NCCN Guidelines for hepatocellular carcinoma, lesions 3 to 5 cm may be treated to prolong survival using arterially directed therapies, or with combination of an arterially directed therapy and ablation as long as tumor location is accessible for ablation. Compared to hepatocellular carcinoma, the vast majority of hepatic PEComa below 5 cm were benign (6). We considered whether radiofrequency ablation could be applied to hepatic PEComa. In another cases, the efficacy of radiofrequency ablation has been reported in benign PEComa below 5 cm (38). We performed a subgroup analysis of patients with tumors less than 5 cm in size. A total of 17 patients were included in the subgroup, including 9 patients in the surgery group, 2 patients in the conservative follow-up group, and 6 patients in the radiofrequency ablation(RFA) group. There was no significant difference between the surgery group and RFA group in gender (Female%,77.8% vs. 66.7%,P>0.05), age (50.7 ± 10.8years vs. 48.33 ± 13.8years,P>0.05) and size(3.29 ± 0.88cm vs. 3.33 ± 0.60cm,P>0.05). During the follow-up period (range, 5–81 months), none of the 15 patients had signs of metastasis or recurrence. This suggests that both surgery and radiofrequency ablation were effective in hepatic PEComa less than or equal to 5 cm, but radiofrequency ablation could be considered as deinitive treatment with less invasion in the context of a multidisciplinary review. The only one patient with a tumor size of 14.6cm could not be surgically resected due to CTPV. However, after multiple treatments of TACE, the tumor shrank and showed internal necrosis, and remained stable during the follow-up of 18 months. Therefore, when patients with PEComa cannot tolerate general anesthesia or cannot achieve surgically resection for other reasons, we can choose appropriate treatment methods according to the actual situation of the tumors (12).

We should acknowledge some limitations of the study. First, due to the rarity of hepatic PEComa, this was a single-center, retrospective study with a small sample size. We need further multicenter, larger-cohort studies to obtain more definitive evidence. Second, the prognosis of patients with malignant tumors in this study is poor. It is necessary to find a better treatment modalitiy and further evaluate its therapeutic effect. Third, because of the heterogeneity in the tumors, the enhanced assessment of radiographic blood flow is subjective, which might lead to bias.

## 5 Conclusion

Hepatic PEComa is a rare mesenchymal tumor that occurs mainly in female. Most of the tumors are benign with non-specific clinical features and imaging findings. The correct diagnosis of PEComa mainly depends on pathological features and immunohistochemistry. Surgery is the best treatment at present, and other treatment methods such as radiofrequency ablation and TACE can also be considered in contraindications of surgery. Long-term clinical follow-up is recommended due to the aggressive behavior and recurrence.

## Data Availability

The original contributions presented in the study are included in the article/supplementary material. Further inquiries can be directed to the corresponding author.
